# Irisin and Its Role in Postmenopausal Osteoporosis and Sarcopenia

**DOI:** 10.3390/biomedicines12040928

**Published:** 2024-04-22

**Authors:** Irene Falsetti, Gaia Palmini, Simone Donati, Cinzia Aurilia, Teresa Iantomasi, Maria Luisa Brandi

**Affiliations:** 1 Department of Experimental and Clinical Biomedical Sciences, University of Florence, 50139 Florence, Italy; irene.falsetti@unifi.it (I.F.); simone.donati@unifi.it (S.D.); cinzia.aurilia@unifi.it (C.A.); teresa.iantomasi@unifi.it (T.I.); 2 Fondazione Italiana Ricerca Sulle Malattie dell’Osso (F.I.R.M.O Onlus), 50129 Florence, Italy; gaia@fondazionefirmo.com

**Keywords:** sarcopenia, postmenopausal osteoporosis, irisin

## Abstract

Menopause, an extremely delicate phase in a woman’s life, is characterized by a drop in estrogen levels. This decrease has been associated with the onset of several diseases, including postmenopausal osteoporosis and sarcopenia, which often coexist in the same person, leading to an increased risk of fractures, morbidity, and mortality. To date, there are no approved pharmacological treatments for sarcopenia, while not all of those approved for postmenopausal osteoporosis are beneficial to muscles. In recent years, research has focused on the field of myokines, cytokines, or peptides secreted by skeletal muscle fibers following exercise. Among these, irisin has attracted great interest as it possesses myogenic properties but at the same time exerts anabolic effects on bone and could therefore represent the link between muscle and bone. Therefore, irisin could represent a new therapeutic strategy for the treatment of osteoporosis and also serve as a new biomarker of sarcopenia, thus facilitating diagnosis and pharmacological intervention. The purpose of this review is to provide an updated summary of what we know about the role of irisin in postmenopausal osteoporosis and sarcopenia.

## 1. Postmenopausal Osteoporosis and Sarcopenia

Menopause is an extremely important phase in women’s lives, involving a series of physical and psychological changes. It occurs because of the inhibition of estrogen secretion.

It is now accepted that the menopause is associated with the onset of bone and muscle diseases, such as osteoporosis and sarcopenia [1,2,3].

Osteoporosis is a chronic skeletal disorder characterized by loss of bone mass and deterioration of bone micro-architecture and consequent increased risk of fractures. Two categories of osteoporosis have been identified: primary and secondary osteoporosis.

Primary osteoporosis is the most common form and includes postmenopausal osteoporosis and senile osteoporosis [1]. Decreased estrogen levels leads to increased apoptosis of osteoblasts, promoting at the same time osteoclast maturation and activity [4]. This results in the loss of the balance between bone resorption and formation, in favor of resorption. In addition, the drop in estrogen leads to an increase in the secretion of cytokines (i.e., interleukin-(IL) 6) closely linked to oxidative stress and inflammation but also to osteoclastogenesis [5,6]. All these factors lead to the onset of postmenopausal osteoporosis.

Sarcopenia is a disease caused by progressive loss of muscle mass and strength resulting in increased fractures and falls. 

Sarcopenia is closely related to menopause [2]. Recent studies reported that estrogen promotes not only the activation and proliferation of satellite cells through its receptors, but also promotes their differentiation, maintaining the balance between synthesis and protein degradation [2,7]. In addition, it inhibits the release of proinflammatory cytokines, which alter muscle proteins, which if damaged, are no longer able to repair the damage to muscle tissue [8]. For these reasons, the drop in estrogen levels during menopause determines the loss of muscle mass and strength.

**Figure 1:**
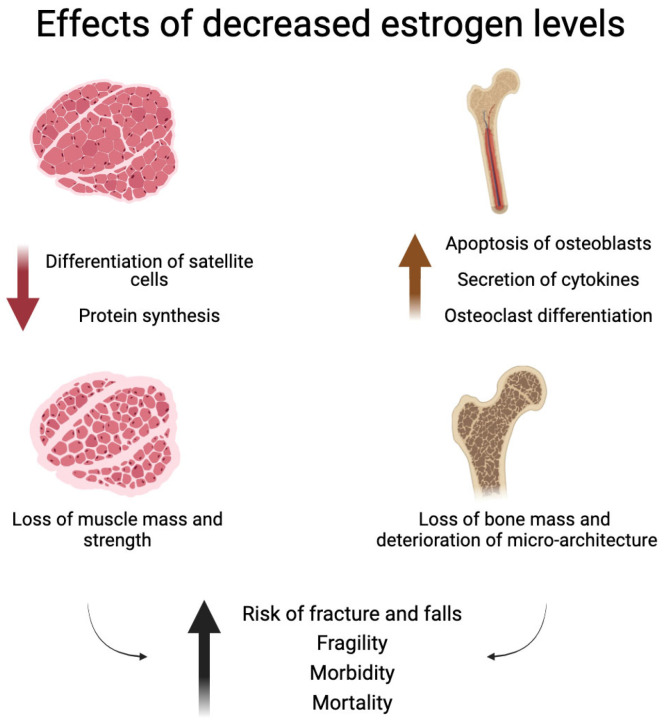
Effects of decreased estrogen levels on bone and muscle. This image was created with BioRender software (https://www.biorender.com/).

The effects of estrogen decline on bone and muscle are summarized in Figure 1.

Postmenopausal osteoporosis and sarcopenia often coexist in the same subject, underlining the stressful link between bone and muscle. This pathological condition is called osteosarcopenia [9]. Osteosarcopenic patients have a higher risk of fracture than those suffering from only one of these conditions and often have an increased mortality once fractured [10].

In fact, osteosarcopenia represents an important social problem with an increase in morbidity and worsening quality of life in those affected, and its incidence has increased in recent years with the increase in the average age of the population [11,12].

For these reasons, it is appropriate study the cellular and molecular mechanisms involved in osteoporosis and sarcopenia to find new therapeutic strategies and therapies. Although to date there are drugs for the treatment of osteoporosis that do not always have beneficial effects on the muscle (hence the importance of research), no drug has been approved for the treatment of sarcopenia [13]. The only non-pharmacological strategy that these two diseases have in common is exercise as it improves general health and prevents or delays their onset and development, while at the same time improving muscle strength and bone mass [14,15,16].

Since it has been observed that the incidence of falls is higher in postmenopausal women than in men of the same age, probably as a consequence of decreased estrogen levels on postural stability and potentially muscle strength, exercise is a good strategy to increase muscle strength, resulting in improved posture and balance [17,18].

Numerous studies in both ovariectomized (OVX) mice and women with postmenopausal osteoporosis have shown that exercise reduces bone mineral loss, improves bone microstructure, and static and dynamic balance with increased muscle strength in the upper and lower limbs [18,19]. Nevertheless, it should not be forgotten that patients affected by osteosarcopenia with a very low bone mass and by sarcopenia are fragile and polymorbid, which makes the applicability of exercise in clinical practice complicated and unattainable in severe cases [20]. For this reason, the search for effective drugs for this pathology is a very important therapeutic field.

Several studies demonstrated that during and after exercise, skeletal muscle fibers secrete biologically active molecules, called myokines, which have been started to be correlated with the bone tissue and not only to skeletal muscle. They represent an interesting starting point to study their activity in terms of possible new targets to find and develop new therapeutical approaches for postmenopausal osteoporosis, sarcopenia, and consequently for osteosarcopenia. Therefore, the aim of this review is to summarize what we know to date about the role of myokines, and in particular on one of them, irisin, on these two pathologies because it seems that they could really function as possible biomarkers for these pathological conditions. In addition, their functions could represent the starting point for the development of new and more effective therapeutical strategies, in particular for sarcopenia.

For this review, we performed a literature search using the PubMed/MEDLINE database with a combination of the keywords myokines and osteoporosis (studies concerning irisin in postmenopausal osteoporosis were included; those concerning the effect of other myokines in postmenopausal osteoporosis were excluded) and myokines and sarcopenia (only studies concerning the study of irisin in sarcopenia were included; those concerning all other myokines in sarcopenia were excluded). All relevant studies were selected and reviewed.

## 2. Myokines

Bone and muscle are intimately connected in a biomechanical crosstalk in which bone is the binding site for muscle and muscle provides the forces necessary for bone to ensure movement. In recent years, however, the importance of a biochemical bone–muscle crosstalk has increasingly emerged with the discovery of the muscle’s ability to secrete substances called myokines, leading to consider the skeletal muscle as an endocrine organ. The secretion of myokines by myocytes and of osteokines (i.e., osteocalcin (*OCN*), osteoprotegerin, fibroblast growth factor (FGF) 23, sclerostin, and receptor activator of nuclear factor κB ligand (RANKL)) by osteocytes represents the way in which muscle and bone send biochemical signals to each other (stimulus or inhibition), thus influencing each other’s metabolism [21].

Myokines are cytokines or peptides synthesized, expressed, and released by skeletal muscle fibers. They exert not only autocrine or paracrine effects by acting on the muscle fibers responsible for their secretion or on neighboring tissues, respectively, but also play an endocrine role acting on tissues far from the site of their secretion [22]. To date, numerous myokines have been identified (i.e., myostatin, IL-6, IL-7, irisin, leukemia inhibitory factor, insulin-like growth factors, FGF2, and brain-derived neurotropic factor (BDNF)), and their biological effects have been studied in the last decades [23].

Among the variety of myokines, we choose to focus on irisin not only because it is one of the latest discoveries, but also because acts as a communication point between bone and muscle, contemporary preventing the loss of bone micro-architecture and muscle mass. In fact, irisin levels were found to be positively correlated with bone mass but also with strength and muscle mass.

These characteristics make irisin an extremely interesting molecule because it preserves muscle function while at the same time exerting a protective action on bone and could therefore be an excellent therapeutic strategy in the prevention and treatment of osteoporosis and sarcopenia [24].

Irisin, discovered in 2012, is a myokine of 112 amino acids that is released following exercise. In fact, exercise results in an increase in the expression of peroxisome proliferator-activated receptor (PPAR)-γ co-activator (PGC)-1α and subsequent expression of a membrane protein called fibronectin type III domain containing (FNDC) 5. The latter, expressed in the brain and muscle, undergoes a proteolytic cleavage to produce irisin [25].

In muscles, irisin promotes myogenic differentiation and myoblast fusion, induces hypertrophy and improves muscle regeneration by up-regulating myocyte growth genes. These effects are attributed to the ability of irisin to activate satellite cells and increase protein synthesis [26].

In bone, irisin stimulates the proliferation and differentiation of osteoblasts through the mitogen-activated protein kinase signaling pathway, inhibiting pyrin domain containing protein 3 (NLRP3) inflammasome and stimulating nuclear factor erythroid 2 related factor 2 (Nrf2) [27]. By blocking nuclear factor-kB and RANKL/nuclear factor of activated T cells type c1, it inhibits osteoclast differentiation [27]. It also increases the vitality of osteocytes [28].

Irisin also acts on adipose tissue by stimulating the browning of white adipose tissue. In addition, it promotes the activity of β-cells, decreasing fasting glucose levels by increasing insulin sensitivity and glucose uptake in liver, muscle, and adipose tissue [29]. Thanks to these properties, irisin can also play an interesting role in many metabolic diseases, such as diabetes and obesity.

**Figure 2:**
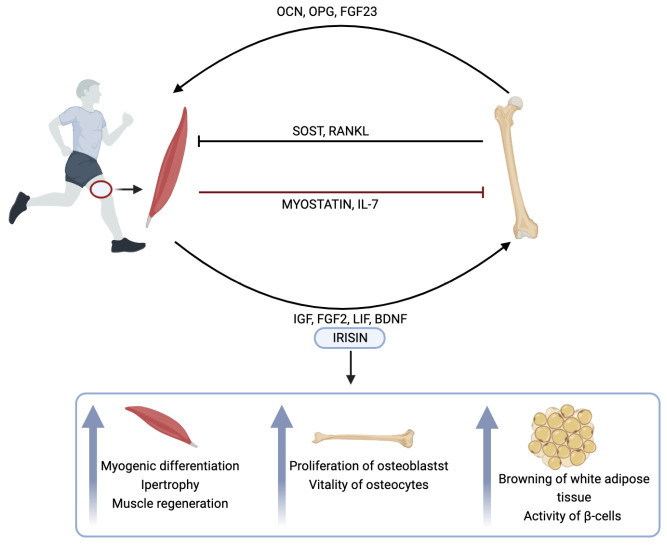
Crosstalk between bone and muscle through cytokine and myokine synthesis, with a focus on the role of irisin in muscle, bone, and adipose tissue. OCN: osteocalcin; OPG: osteoprotegerin; FGF: fibroblast growth factor; SOST: sclerostin; RANKL: receptor activator of nuclear factor κB ligand; IL-7: interleukin-7; IGF: insulin-like growth factor 1; LIF: leukemia inhibitory factor; BDNF: brain-derived neurotropic factor. This image was created with BioRender software (https://www.biorender.com/).

Figure 2 shows the crosstalk between bone and muscle and summarizes the effects of irisin on muscle, bone, and adipose tissue.

## 3. Irisin and Its Role in Postmenopausal Osteoporosis

Several studies have evaluated levels of irisin in women with postmenopausal osteoporosis and in animal models. Regarding the latter, rats undergoing ovariectomy are a good model for studying postmenopausal osteoporosis. Of note, 14 days after surgery, significant bone loss was observed in the proximal tibial metaphysis; after one month, the distal femur seemed to be particularly susceptible to bone loss, which progressively increased over time. In fact, after 36 weeks, bone loss reaches approximately 57/64% in relation to the spine [30]. The decrease in bone mineral density (BMD) is also associated with a reduction in bone perfusion.

Administration of irisin to Sprague-Dawley rats with postmenopausal osteoporosis resulted in improved BMD, trabecular thickness, trabecular number, and inhibition of osteoblast apoptosis [31]. Irisin resulted in increased expression levels of *runt-related transcription factor 2*, *OCN*, *Bcl-2*, and *Nrf2* and decreased expression levels of *caspase 3* (*CASP3*) and *NLRP3*. The authors conclude that irisin, by up-regulating *Nrf2* and inhibiting *NLRP3* expression, can be used to treat postmenopausal osteoporosis.

Kawao et al., evaluated the effects of chronic treadmill exercise of moderate intensity on muscle and bone in OVX and sham-operated mice [32]. OVX caused a decrease in cortical and trabecular BMD, bone mineral content, and femoral thickness and area. Exercise significantly improved cortical and trabecular BMD of the femur in OVX mice, proving to be an excellent strategy to prevent postmenopausal osteoporosis. Higher levels of *Fndc5* mRNA and irisin protein were recorded in the gastrocnemius and soleus muscles in both OVX and non-OVX mice following exercise (this agrees with the results of the study by Iemura et al., where it is observed that OVX alone does not affect *Fndc5* mRNA levels in gastrocnemius and soleus muscles [33]). In gastrocnemius muscle, regression analysis revealed that *Fndc5* mRNA levels were positively correlated with trabecular BMD of femurs and tibias. In addition, a significant increase in *Fndc5* mRNA levels was demonstrated in the femurs of OVX mice. These results indicate that the positive effects of chronic exercise on bone can be correlated to increased *irisin* expression levels in OVX mice, suggesting a potential role for irisin as a biomarker in the prevention and treatment of osteoporosis.

A recent study reported that the intraperitoneal administration of recombinant irisin (100 µg/kg twice a week for 5 weeks) in OVX mice prevented trabecular bone loss and resulted in a significant increase in BMD, bone to tissue volume ratio, connection density, and number of trabeculae compared with saline-treated OVX mice [34]. Irisin also induced a significant increase in the number of osteoblasts and a significant decrease in the number of osteocytes on the trabecular surface. This agrees with increased serum levels of OCN (a biomarker of bone formation) and decreased serum levels of tartrate-resistant acid phosphatase (TRAP, related to bone resorption). Overall, these results indicate that irisin prevents bone loss and improves bone quality in OVX mice as it establishes a new balance by increasing the number of osteoblasts and decreasing that of osteoclasts, paving the way for the possible therapeutic use of irisin in postmenopausal osteoporosis.

In the study by Morgan et al., serum levels of bone markers (OCN, bone alkaline phosphatase, TRAP), calcium, and phosphorus were measured, and a significant decrease in these parameters was observed in OVX rats treated with irisin (100 µg/kg/week for 4 weeks) compared to OVX rats [30]. Irisin can restore these parameters almost completely, returning them to the levels measured in the control group, the sham-operated group, and the OVX group. Histological analysis of the distal femoral diaphysis of OVX rats revealed the loss of normal bone structure with resorbed bone cavities and increased numbers of osteoclasts. Conversely, in the irisin-treated OVX rats, there were few resorbed bone cavities, few osteoclasts, and many osteocytes. The authors conclude that irisin treatment in OVX rats prevents bone structure loss, proving to be a possible candidate for the treatment of postmenopausal osteoporosis.

A reduction in circulating levels of irisin has been found in women with postmenopausal osteoporosis [35]. In addition, an inverse correlation was observed between circulating irisin levels and the presence of previous osteoporotic fractures in postmenopausal women, independent of 25(OH)-vitamin D levels and bone markers [36,37]. In both of these works, no correlation was found between levels of irisin and BMD or with lean mass. According to the authors, irisin may have a protective action on the bone regardless of BMD [37].

However, a positive correlation between irisin levels and BMD has been observed in other studies. The aim of Lu et al.’s work is to evaluate the presence of a correlation between irisin levels and BMD in maintenance hemodialysis (MHD) patients, who were divided into three groups: osteoporotic, osteopenic, and control [38]. In the first two groups, where the average age was higher and there was a prevalence of the female sex, lower levels of irisin and body mass index (BMI) were found to correlate positively with lumbar BMD. The authors argue that irisin can be used as a bone marker in MHD patients because it may ameliorate the osteoporosis induced by muscle disuse that can occur in patients with chronic diseases.

Another group investigated the correlation between levels of irisin and BMD in older men with osteoporosis and osteopenia. In this case, too, irisin levels were lower than the control group, correlated positively with BMD, and were independent of BMI and 25(OH)-vitamin D. The authors argue that irisin has a protective action on bone [39].

Lower levels of irisin were also found in postmenopausal women with minimal hip fractures (MTHF) and positively correlated with BMD. This is an interesting study because according to the authors irisin could act as a predictor of the risk of MTHF in older women [40].

In addition to demonstrating a weak positive correlation between irisin and BMD, Zhou et al., showed that myokine levels were lower in postmenopausal women and fracture patients than in osteoporotic patients [41]. Even in 80 women with postmenopausal osteoporosis, serum levels of irisin were significantly lower than in the control group [42]. These correlated positively with the BMD and serum human C terminal telopeptides of types I collagen (marker of bone resorption) but negatively with the T-score and serum human carboxy-terminal propeptide of type I procollagen (marker of bone resorption). Since irisin is closely related to bone turnover markers, the authors conclude that irisin could be used in the prevention, diagnosis, and treatment of postmenopausal osteoporosis.

Liu et al., found significantly lower circulating serum levels of irisin in postmenopausal women with a hip fracture than in postmenopausal women without fractures [43]. These correlated positively with both total body and hip BMD. Furthermore, a low circulating irisin level was associated with a high risk of osteoporosis and fractures. The authors conclude that irisin may therefore be a valuable tool in the prevention and treatment of osteoporosis and fractures.

Colaianni et al., evaluated the role of irisin in muscle and bone in elderly subjects with osteoporosis or osteopenia undergoing total hip or knee replacement, and this study is extremely important because they assessed any correlations of irisin with data obtained from muscle and bone biopsies within the same population [44]. Serum irisin levels correlated negatively with patient age and positively with BMD of the femur and hip. In addition, not only was a positive correlation observed between *Fndc5* expression in muscle biopsies and *OCN* mRNA, but also between the number of Fndc5-positive fibers in muscle biopsies and BMD of the femur and femoral neck, thus demonstrating that *irisin* expression in skeletal muscle is associated with improved bone mass. Because lower serum levels of irisin were found in patients with osteopenia/osteoporosis than in healthy controls, the authors assessed the existence of a correlation between this reduction and cellular senescence by studying the expression of the senescence marker p21 in both bone and muscle. Increased *p21* expression levels were observed in the bone of osteoporotic patients compared with healthy subjects, but not in muscle biopsies. Furthermore, they observed in vitro that irisin inhibited *p21* expression in osteoblasts after 8 h of treatment. It has been demonstrated that the observed inhibitory effect is specific because irisin-induced downregulation was attenuated by the use of an irisin-specific neutralizing antibody. The authors state that by witnessing a decrease in *irisin* levels and an increase in p21 levels in osteoporosis, irisin could be a therapeutic strategy to delay osteoporosis.

Overall, all the described results indicate that irisin may exert a protective role on bone and be considered a prognostic biomarker for osteoporosis.

In Table 1, we have summarized the main effects of irisin in postmenopausal osteoporosis.

## 4. Irisin and Its Role in Sarcopenia

As there is no specific biomarker of sarcopenia available to date, the study by Park et al., aimed to investigate the existence of a link between irisin and sarcopenia and to evaluate irisin as a possible biomarker of sarcopenia in postmenopausal women [45]. They showed that circulating irisin levels were significantly lower in sarcopenic patients but also that a serum irisin concentration of less than 1 ng/mL carries a 95% risk of developing sarcopenia. The authors state that low levels of this myokine are closely linked to sarcopenia in postmenopausal women and can therefore be used as a biomarker in the early diagnosis of sarcopenia.

The purpose of Yen et al.’s work was also to identify a biomarker useful in the diagnosis of sarcopenia by analyzing coenzyme Q10, creatine kinase, albumin, irisin, myostatin in sarcopenic and non-sarcopenic patients [46]. Significant differences between the two groups were found only in levels of irisin and creatine kinase, although about half of the patients had a low concentration of coenzyme Q10. They attributed the increased ability to predict sarcopenia to irisin and creatine kinase. In addition, low levels of coenzyme Q10, irisin, and creatine kinase are linked to an increased risk of sarcopenia. Altogether, these three can be considered biomarkers of sarcopenia.

Other studies have also shown that sarcopenic or pre-sarcopenic patients are characterized by lower circulating levels of irisin than healthy subjects [45,47,48].

In their study, Chang et al., evaluated irisin in a group of 715 subjects divided into three groups: pre-sarcopenia, sarcopenia, and control [47]. Irisin levels were significantly lower in the first two groups, indicating a strong association between irisin and sarcopenia. The authors state that irisin could be used in the prevention and onset of muscular atrophy and as a marker of sarcopenia ensuring early diagnosis.

In the cross-sectional study conducted by Alsaawi et al., on elderly women with or without sarcopenia, the indices of BMI, mid-arm muscle area, mid-arm circumference, abdominal volume index, and levels of irisin were lower in sarcopenic patients, confirming a strong link between sarcopenia and anthropometric measurements [48]. No association was found between sarcopenia and protein intake. Further investigation may be needed to assess the influence of diet on the onset of sarcopenia.

Zhou et al., measured circulating levels of irisin in rats undergoing orchiectomy [49]. In the latter, irisin levels were significantly lower than in rats undergoing sham operation. The authors claim that irisin could therefore be used as a biomarker and therapeutic target for sarcopenia in men, as it is involved in the pathogenesis of this disease.

In sarcopenic women and men, Wang et al., explored the existence of a relationship between irisin and vitamin D levels, the deficiency of which appears related to reduced muscle mass, function, and strength [50]. They observed a positive relationship between 25(OH)D and 25(OH)D_3_ levels and irisin levels in sarcopenic women but not in men. This difference appears to be due to women’s greater subcutaneous fat mass and the action of estrogen. The authors state that preventive vitamin D supplementation would keep irisin levels high to slow down the onset of sarcopenia. Nevertheless, not all the studies reported a correlation between irisin and sarcopenia.

In their work, Choi et al., reported that sarcopenia is more common in women (42.1%) than in men (13.4%), but they did not find any difference in circulating irisin levels between sarcopenic and non-sarcopenic patients in both study groups [51].

Also, in the study conducted by Baek et al., no correlation was found between circulating irisin levels and the state of sarcopenia, low muscle mass, weak muscle strength, poor physical performance, or poor muscle quality among sarcopenic and non-sarcopenic patients [52]. However, the authors state that the lack of correlation between sarcopenia and circulating irisin levels can be attributed to two factors. The first factor is that to date there is no clear definition of sarcopenia and no common assessment method, so it would be desirable to provide a comprehensive definition of sarcopenia that includes multiple muscle parameters and accurate measurement methods. The second reason could be attributed to the diversity of patients with sarcopenia, as a distinction must be made between patients with obesity and good nutritional status and those with malnutrition and cachexia. In fact, the authors surmise that the lack of correlation between irisin levels and sarcopenia in their work and in Choi’s work could depend on the very characteristics of the patients (relatively lower BMI compared to patients without sarcopenia) and that further investigations are needed because irisin could also be useful in recognizing a certain type of sarcopenia [51].

As there are numerous molecular mechanisms involved in the pathogenesis of sarcopenia, Qaisar et al., evaluated 6 circulating biomarkers in healthy and diseased male patients (chronic heart failure (CHF) or chronic obstructive pulmonary disease (COPD)) diagnosed with sarcopenia [53]. The purpose of the work is to develop a biomarker panel for sarcopenia covering more than one physiopathological mechanism. The selected biomarkers are related to skeletal muscle metabolism, growth, regeneration, and systemic inflammation and are c-terminal agrin fragment 22 (*CAF22*), pro-peptide amino-terminal of type III procollagen (*P3NP*), *osteonectin*, irisin, fatty acid-binding protein 3 (*FABP3*), and macrophage migration inhibitory factor (*MIF*). Levels of *P3NP*, *CAF22*, *osteonectin*, *FABP3* and *MIF* were found to be higher in patients with COPD and CHF with advanced sarcopenia than in healthy controls, while those of irisin were lower. The authors state that these can be considered sarcopenia biomarkers and are developing a biomarker panel, which represents an interesting instrument for the diagnosis and evaluation of sarcopenia.

It has been observed that sarcopenia in COPD leads to a worsening of physical performance in functional tests but also a decrease in the strength of the respiratory muscles, and its occurrence seems to be due to oxidative stress and inflammation. Lage et al., examined levels of irisin, BDNF, and tumor necrosis factor-α (TNF-*α*) in sarcopenic patients with COPD, where lower levels of the two myokines and higher soluble TNF-*α* receptors were found [54]. These data correlate with a decrease in the strength of the respiratory muscles. The authors state that these data can help in the prevention of sarcopenia in patients with COPD, being the first study to evaluate the levels of these two myokines and TNF-*α* receptors, body composition, and anthropometry by assessing respiratory muscle strength in COPD.

According to D’Amuri et al., muscle–fat tissue crosstalk could represent a new biological target for the treatment of sarcopenia and muscle disuse atrophy [55]. Fourteen days of bed rest resulted in a loss of both mass and function in muscle but an increase in body fat tissue mass. However, an increase in circulating levels of irisin following bed rest was found, which was attributed to the ability of fat tissue to synthesize and release irisin. Interestingly, in subjects in whom irisin levels were increased, muscle damage was reduced; thus, the ability to respond to the damage caused by bed rest was greater. Hence, the authors proposed the existence of a negative feedback loop in the interactions between muscle and adipose tissue, in which the muscle acts on the adipose tissue by synthesizing irisin during exercise and the adipose tissue defends the muscle during inactivity by increasing irisin synthesis.

Guo et al., evaluated differences in Fndc5/irisin levels in mouse skeletal muscles associated with the aging process [56]. In mice aged 24 months, *Fndc5* mRNA expression was reduced in the skeletal muscles of the hind limbs, femoral quadriceps, gastrocnemius, and anterior tibial versus young controls. By analyzing the gastrocnemius, they observed a decrease in the levels of proteins Fndc5 and irisin. The influence of Fndc5/irisin deficiency on sarcopenia in 22-month-old Fndc5/irisin knockout mice was evaluated. This has led to a sharp worsening of muscle atrophy associated with aging (observed a reduction in the weight of the gastrocnemius, anterior tibial muscles, and femoral quadriceps; increases in the levels of atrophic and inflammatory genes and protein levels of ubiquitin ligase atrophic MAFbx and Murf-1; and decreases in the grip force and size of the fibers). They then administered the recombinant irisin protein in a prevention model (2 mg/kg to 14-month-old mice for 4 months) and in a treatment model (2 mg/kg to 22-month-old mice for one month). In both models, the administration of irisin resulted in a marked improvement in sarcopenia, confirmed by increased grip strength, muscle weight (greater in the gastrocnemium) and muscle fiber size, and reduced mRNA levels of atrophic and inflammatory genes and MAFbx and Murf-1 protein levels. These results are very interesting because they show that irisin could be used to treat aging-related sarcopenia in mice by restoring muscle function.

Patients with myotonic dystrophy have sarcopenia. Dozio et al., evaluated circulating levels of irisin in patients with type 1 and 2 myotonic dystrophy compared to healthy subjects [57]. Levels of irisin were lower in patients with myotonic dystrophy than in healthy controls but no differences were found in the release of irisin from the myostatic tubes of patients and control patients. The authors therefore assume that the decrease in circulating levels of irisin can be attributed to the reduction in skeletal muscle mass affecting these patients rather than to real endocrine damage as muscle fibers continue to secrete irisin. The authors suggest that the use of recombinant irisin in these patients could have a positive effect on their functional and metabolic profile.

Wu et al., demonstrated that irisin can be used in the treatment of sarcopenia by evaluating the effects of irisin in D-galactose-induced skeletal muscle fibroblasts [58]. *Irisin* or *Fndc5* over-expression inhibited senescence and skeletal muscle fibrosis and improved redox balance. They restore the redox system by increasing the activation and subsequent phosphorylation of Nrf2 and the activity of antioxidant enzymes (glutathione and superoxide dismutase), but also by decreasing the production of reactive oxygen species and the expression of malondialdehyde and NADPH oxidase 4. These beneficial effects of irisin have been attributed to its ability to activate the phosphatidylinositol 3-kinase/protein kinase B signaling pathway.

The protective effect of irisin in fibrosis has also been demonstrated in a mouse model of muscular dystrophy [59]. Administration of irisin (2.5 μg per gram of body weight three times per week for two weeks) in 6-week-old adult mice decreased fibrosis in tibialis anterior muscle by about four-fold and necrotic muscle fibers by 43%. Taken together, these data indicate that irisin can protect against fibrotic tissue accumulation and necrosis of myofibers. In addition, prolonging the duration of irisin treatment (three times a week for 4 weeks) in 4-week-old mice resulted in an approximately two-fold increase in the grip strength of the forelimbs and the weight of tibialis anterior, soleus, and gastrocnemius skeletal muscles. Overall, irisin was shown to be effective in improving muscle function and increasing muscle mass due to its pro-myogenic effect.

Demir et al., measured levels of irisin and TNF-α in patients with cancer, sarcopenia, and non-sarcopenic individuals [60]. In sarcopenic patients, serum levels of irisin and TNF-α were significantly lower and higher than those noted in non-sarcopenic individuals, respectively, and these markers could be independent predictors of sarcopenia. According to the authors, irisin could be used in the diagnosis of sarcopenia, thus preventing its consequences in subjects with cancer, while TNF-α appears to play a role in its pathophysiology by inhibiting protein synthesis in muscle cells. Currently, this is the first work that analyses the levels of irisin and TNF-α together in sarcopenic patients with cancer, and it is interesting because the inflammation present in many types of cancer can promote the onset of sarcopenia. This is a very important field of investigation because preventing and recognizing sarcopenia in these patients could allow maintenance of physical strength and increase compliance to treatment.

Oflazoglu et al., also investigated irisin levels in cancer patients, specifically newly diagnosed non-metastatic colorectal cancer patients, and found a negative correlation between irisin and sarcopenia, evidence of a possible link between inflammation and sarcopenia [61]. As both sarcopenia and cancer have become more common in recent years, it may be useful to evaluate levels of irisin in larger groups with different types of cancer to establish an association among irisin, sarcopenia, and cancer.

Lee et al., investigated the action of irisin on sarcopenia and cardiovascular disease in patients in peritoneal dialysis (PD) [62]. Irisin levels were lower than the controls. The authors showed that irisin was predictive for carotid atherosclerosis in PD patients; however, further investigation is needed to establish the mechanism behind this connection.

The purpose of Wu et al.’s work was to generate a model that could predict sarcopenia in patients undergoing PD [63]. The characteristics examined are grip strength, BMI, total body water value, irisin, extracellular water/total body water, fat-free mass index, phase angle, albumin/globulin, blood phosphorus, total cholesterol, triglyceride, and prealbumin. The authors argue that the model, having successfully predicted sarcopenia, could be used as a screening tool. This, in the future, could prove very useful given that to date the instrumentation used in diagnosis of sarcopenia is very demanding and expensive.

The purpose of de Luis et al.’s work is to assess levels of irisin and myostatin in patients suffering from disease-related malnutrition (DMR) with or without sarcopenia [64]. It is particularly interesting because irisin and myostatin have opposite effects: irisin stimulates protein synthesis, while myostatin inhibits it. Patients without sarcopenia had higher levels of irisin and were stronger. There were no differences in myostatin levels between the two groups. In addition, an association with the parameters of strength and muscle mass was detected only for irisin. Therefore, the authors claim that irisin is closely associated with sarcopenia in patients with DMR and that it could be used as a biomarker for the diagnosis or treatment of these patients. It might be useful to investigate further the levels of these two myokines in larger patient populations in order to assess whether the balance in their levels is lost over the years in favor of myostatin, which could therefore be connected with the decrease in strength and muscle atrophy.

Myostatin and irisin levels were also analyzed in patients with type 2 diabetes mellitus with or without sarcopenia, as the incidence of sarcopenia increases in patients with diabetes [65]. Also, in this study, myostatin levels did not differ between the two groups (the authors hypothesize that this can be attributed to the low number of participants), while irisin levels were lower in sarcopenic patients. The authors argue that a risk factor for the onset of sarcopenia is low levels of irisin but also inadequate blood sugar control.

In order to establish the relationship between sarcopenia and liver disease, Zhao et al., evaluated serum levels of irisin in sarcopenic patients with liver cirrhosis [66]. No differences were found in etiology, albumin levels, total cholesterol and very low-density lipoprotein, glucose, and insulin among patients with cirrhosis and with or without sarcopenia. In sarcopenic subjects with cirrhosis, significantly lower levels of irisin were found, and it was observed that these levels were lower following the worsening of liver function reserve. The authors assume that this decrease in levels can be attributed to sarcopenia and cirrhosis of the liver but also that the elimination of irisin occurs mainly through the kidney. In this study, it was shown for the first time that irisin is an independent parameter associated with sarcopenia in patients with cirrhosis. However, further studies are needed to clarify the role of irisin because sarcopenia in these patients has a high incidence and involves infections, hepatic encephalopathies, and longer hospital stays with worsening quality of life and increased health costs.

Boga et al., also evaluated irisin and myostatin levels in sarcopenic patients with chronic liver disease [67]. Lower levels of irisin and higher levels of myostatin were recorded in sarcopenic patients. The values of both myokines were found to be independent predictors of sarcopenia; however, those of myostatin were better correlated with the severity of liver disease. In fact, the authors claim that myostatin can be a predictor of sarcopenia in all stages of cirrhosis.

Kukla et al., also examined irisin levels in patients with decompensated advanced chronic liver disease [68]. There were no differences in serum levels of irisin between cirrhotic patients as well as between cirrhotic patients with or without diabetes and those who were overweight or not. No association was found between irisin and severity of liver cirrhosis. The authors speculate that this absence of a difference may be due to liver disease. In fact, levels of irisin may vary in relation to immobilization associated with the current complication, bleeding from esophageal varicose veins, or infection. From this work clearly emerges the need to identify a marker for sarcopenia in patients with cirrhosis of the liver and the effort that needs to do research in this field in the coming years.

Taken together, these results indicate that irisin as a pro-myogenic factor could be used in the treatment of sarcopenia triggered by various causes.

In Table 2, we have summarized the main effects of irisin in sarcopenia.

## 5. Conclusions

Menopause represents a very delicate stage in the life of a woman and is accompanied by numerous changes. The decrease in estrogen that occurs during menopause is associated with the occurrence of numerous diseases, such as osteoporosis and sarcopenia. To date, there are drugs available for osteoporosis which, however, do not always have beneficial effects on muscles, while no drug has yet been approved for the treatment of sarcopenia or fractured osteosarcopenic patients. The non-pharmacological management of these two pathologies includes regular physical exercise, during which myokines are released from the muscles. Myokines have important biological functions in the muscle but also in the bone. Among these myokines, irisin has aroused great interest in recent years as it has been shown to have myogenic properties and an anabolic effect on bone, increasing bone mass and stimulating osteoblastogenesis. It was shown that irisin levels were lower in patients with both postmenopausal osteoporosis and sarcopenia than in healthy subjects, so scientific evidence to date shows that irisin could be used as a biomarker for these two diseases. Furthermore, it could be used in the treatment of postmenopausal osteoporosis as it is able to prevent bone loss and in the treatment of sarcopenia as it resulted in increased muscle mass and improved muscle function. Gaining a deeper understanding of the role and properties of irisin could pave the way for new therapeutic strategies for bone diseases, such as osteoporosis, while also identifying a new biomarker of the muscular state to facilitate diagnosis and consequently the timely initiation of therapy in patients with sarcopenia or muscle diseases. It is an extremely important field of research, not least because these are age-related diseases. In addition, with the increase in the average age of the population, it is desirable to be able to identify new molecules to be used in their diagnosis and treatment.

## Figures and Tables

**Table 1:** Irisin and its role in postmenopausal osteoporosis. BMD: bone mineral density; RUNX2: runt-related transcription factor 2; OCN: osteocalcin; Nrf2: nuclear factor E2-related factor 2; CASP3: caspase 3; NLRP3: NLR family, pyrin domain containing protein 3; OVX: ovariectomized; TRAP: tartrate-resistant acid phosphatase; BAP: bone alkaline phosphatase; MHD: maintenance hemodialysis.

Population	Sample Size	Age (Mean ± SD)	Induction of Osteoporosis	Condition	Effects	Reference
Rats	45	/	OVX	Postmenopausal osteoporosis	Improvements in BMD, trabecular thickness, trabecular number Inhibition of osteoblast apoptosis Increased expression levels of RUNX2, OCN, Bcl-2, and Nrf2 Decreased expression levels of CASP3 and NLRP3	[31]
Mice	36	Eight weeks old	OVX	Postmenopausal osteoporosis	Improvements in cortical and trabecular BMD of the femur Higher levels of irisin protein Increased Fndc5 mRNA levels	[32]
Mice	37	Ten weeks old	OVX	Postmenopausal osteoporosis	Increases in BMD, bone volume to tissue ratio, connection density, and number of trabeculae Increases in the number of osteoblasts and serum levels of OC Decreases in serum levels of TRAP and the number of osteocytes on the trabecular surface	[34]
Rats	40	/	OVX	Postmenopausal osteoporosis	Improvements in serum levels of OCN, BAP, TRAP, calcium, and phosphorous Improvements in bone micro-architecture	[30]
Women	176	63.96 ± 5.98 (control group); 67.92 ± 8.14 (osteoporotic group)	/	Postmenopausal osteoporosis	Lower irisin levels compared to the control group	[35]
Women	125	65.7 ± 1.3	/	Postmenopausal with low bone mass	Inverse correlation between irisin levels and osteoporotic fractures	[36]
Women	72	64.3 ± 6.1	/	Postmenopausal osteoporosis	Inverse correlation between irisin and vertebral fractures	[37]
Women and men	80	66.93 ± 10.27	/	Osteoporotic and osteopenic MHD patients	Lower irisin levels compared to the control Positive correlation with lumbar BMD	[38]
Men	67	63.96 ± 5.98 (control group); 66.20 ± 6.07 (osteopenic group); 67.92 ± 8.14 (osteoporotic group)	/	Osteoporosis and osteopenia	Lower irisin levels compared to the control Positive correlation with BMD	[39]
Women	320	76 (control group); 78 (study group)	/	Minimal trauma hip fractures	Lower irisin levels compared to the control Positive correlation with BMD	[40]
Women and men	1018	>40	/	Osteoporosis	Lower irisin levels in postmenopausal women and with a history of fractures Positive correlation with BMD	[41]
Women	175	58.41±3.51 (control group); 59.73 ± 3.44 (study group)	/	Postmenopausal osteoporosis	Lower irisin levels compared to the control Positive correlation with BMD Negative correlation with T-score	[42]
Women	430	68.7 ± 11.7	/	Hip fractures	Lower irisin levels compared to the control Positive correlation with BMD	[43]
Women and men	62	68.71 ± 12.31	/	Osteoporosis or osteopenia	Lower irisin levels compared to the control Positive correlation with BMD Positive correlation between Fndc5 expression in muscle biopsies and OCN mRNA Increased p21 expression level	[44]

**Table 2:** Irisin and its role in sarcopenia. ORX: orchiectomy; BAT: brown adipose tissue; CHF: chronic heart failure; COPD: chronic obstructive pulmonary diseases; BDNF: brain-derived neurotropic factor; TNF-α: tumor necrosis factor-α; DM: myotonic dystrophies; CD: cardiovascular disease; PDP: peritoneal dialysis patients; DMR: disease-related malnutrition; T2DM: type 2 diabetes mellitus; dACLD: decompensated advanced chronic liver disease.

Population	Sample Size	Age (Mean ± SD)	Induction of Sarcopenia	Condition	Effects	Reference
Women	153	72.20 ± 5.96	/	Postmenopause	Lower irisin levels compared to the control 1 ng/mL of irisin carries a 95% risk of developing sarcopenia	[45]
Women and men	99	74.8 ± 7.4 (sarcopenic group); 72.0 ± 8.4 (non-sarcopenic group)	/	Sarcopenia	Lower irisin levels compared to the control Association between low irisin levels and increased risk of developing sarcopenia	[46]
Women	715	18–90	/	Sarcopenia	Lower irisin levels compared to the control	[47]
Women	131	65.9 ± 5.5	/	Sarcopenia	Lower irisin levels compared to the control	[48]
Rats	20	3 months old	ORX	Androgen deficiency	Lower irisin levels compared to the control	[49]
Women and men	422	66.1-74.1	/	Sarcopenia	A positive relationship between 25(OH)D and 25(OH)D3 levels and irisin levels in sarcopenic women	[50]
Women and men	80; 401	42 (BAT negative); 40 (BAT positive); 61 (sarcopenic group); 52 (non-sarcopenic group)	/	Sarcopenia and BAT	No difference in irisin levels	[51]
Women and men	143	71.83 ± 5.56 (sarcopenic group); 69.01 ± 6.17 (non-sarcopenic group)	/	Sarcopenia	No difference in irisin levels	[52]
Men	258	62.6 ± 5.5 (control group); 64.3 ± 3.7 (COPD group); 66.9 ± 5.4 (CHF group)	/	Sarcopenia and CHF or COPD	Lower irisin levels compared to the control	[53]
Women and men	86	72.7 (non-COPD); 73.9 (COPD group)	/	Sarcopenia and COPD	Lower irisin and BDNF levels Higher levels of soluble TNF-α receptors	[54]
Men	23	23.3 ± 2.8; 59.3 ± 3.0	/	/	Increased irisin levels after bed rest Reduced muscle damage	[55]
Mice	/	14 months old; 22 months old	Ageing	Sarcopenia	Improvement of sarcopenia Decreased mRNA levels of atrophic and inflammatory genes Decreased levels of MAFbx and MURF-1 proteins	[56]
Men	61	45.6 ± 14.5 (control group); 44.7 ± 11.5 (DM1 group); 56.7 ± 9.3 (DM2 group)	/	DM1 and DM2	Lower irisin levels compared to the control	[57]
Skeletal muscle fibroblasts	/	/	D-galactose	Senescence, fibrosis, and redox imbalance	Inhibition of senescence and skeletal muscle fibrosis Improvement of the redox balance	[58]
Mice	/	6 months old	/	Duchenne muscular dystrophy	Decreased fibrosis in anterior tibial muscle and necrotic muscle fibers Increased grip strength of the forelimbs and weight of the anterior tibial skeletal muscles, soles, and gastrocnemius	[59]
Women and men	141	59.84 ± 11 (non-sarcopenic group); 61.46 ± 9.7 (sarcopenic group)	/	Sarcopenia and cancer	Lower irisin levels compared to the control Higher levels of TNF-α	[60]
Women and men	50	60	/	Sarcopenia and cancer	Negative relationship between sarcopenia and irisin	[61]
Women and men	137	54.1 ± 11.5 (control group); 54.1 ± 11.6 (PD group)	/	Sarcopenia and CD in PDP	Lower irisin levels compared to the control	[62]
Women and men	105	53 ± 8.59 (non-sarcopenic group); 57 ± 9.04 (sarcopenic group)	/	Sarcopenia and PDP	The model could predict sarcopenia	[63]
Women and men	108	67.4±3.4	/	Sarcopenia and DMR	Lower irisin levels compared to the control No difference in myostatin levels	[64]
Women and men	90	55.01 ± 8.81 (non-sarcopenic group); 54.17 ± 7.68 (sarcopenic group)	/	Sarcopenia and T2DM	Lower irisin levels compared to the control No difference in myostatin levels	[65]
Women and men	187	58	/	Sarcopenia and liver cirrhosis	Lower irisin levels compared to the control	[66]
Women and men	145	53.4 ± 8.5 (control group); 55.3 ± 10.4 (cirrhosis group)	/	Sarcopenia and liver disease	Lower irisin levels compared to the control Higher levels of myostatin compared to the control	[67]
Women and men	88	57.9	/	Sarcopenia and dACLD	No difference in levels of irisin	[68]

## References

[1] Filippi L., Camedda R., Frantellizzi V., Urbano N., De Vincentis G., Schillaci O. (2023). Functional imaging in musculoskeletal disorders in menopause. Semin. Nucl. Med..

[2] Geraci A., Calvani R., Ferri E., Marzetti E., Arosio B., Cesari M. (2021). Sarcopenia and menopause: The role of Estradiol. Front. Endocrinol..

[3] Kawao N., Kaji H. (2015). Interactions between muscle tissues and bone metabolism. J. Cell Biochem..

[4] Khosla S., Oursler M.J., Monroe D.G. (2012). Estrogen and the skeleton. Trends Endocrinol. Metab..

[5] Lu L., Tian L. (2023). Postmenopausal osteoporosis coexisting with sarcopenia: The role and mechanisms of estrogen. J. Endocrinol..

[6] Kim O.Y., Chae J.S., Paik J.K., Seo H.S., Jang Y., Cavaillon J.M., Lee J.H. (2012). Effects of aging and menopause on serum interleukin-6 levels and peripheral blood mononuclear cell cytokine production in healthy nonobese women. Age.

[7] Collins B.C., Arpke R.W., Larson A.A., Baumann C.W., Xie N., Cabelka C.A., Nash N.L., Juppi H.K., Laakkonen E.K., Sipilä S. (2019). Estrogen regulates the satellite cell compartment in females. Cell Rep..

[8] Li Y.P., Reid M.B. (2000). NF-kappaB mediates the protein loss induced by TNF-alpha in differentiated skeletal muscle myotubes. Am. J. Physiol. Regul. Integr. Comp. Physiol..

[9] Hirschfeld H.P., Kinsella R., Duque G. (2017). Osteosarcopenia: Where bone, muscle, and fat collide. Osteoporos. Int..

[10] Sheng R., Cao M., Song M., Wang M., Zhang Y., Shi L., Xie T., Li Y., Wang J., Rui Y. (2023). Muscle-bone crosstalk via endocrine signals and potential targets for osteosarcopenia-related fracture. J. Orthop. Translat..

[11] Clynes M.A., Gregson C.L., Bruyère O., Cooper C., Dennison E.M. (2021). Osteosarcopenia: Where osteoporosis and sarcopenia collide. Rheumatology.

[12] Coll P.P., Phu S., Hajjar S.H., Kirk B., Duque G., Taxel P. (2021). The prevention of osteoporosis and sarcopenia in older adults. J. Am. Geriatr. Soc..

[13] Dent E., Morley J.E., Cruz-Jentoft A.J., Arai H., Kritchevsky S.B., Guralnik J., Bauer J.M., Pahor M., Clark B.C., Cesari M. (2018). International Clinical Practice Guidelines for Sarcopenia (ICFSR): Screening, Diagnosis and Management. J. Nutr. Health Aging.

[14] Colaianni G., Storlino G., Sanesi L., Colucci S., Grano M. (2020). Myokines and osteokines in the pathogenesis of muscle and bone diseases. Curr. Osteoporos. Rep..

[15] Hettchen M., von Stengel S., Kohl M., Murphy M.H., Shojaa M., Ghasemikaram M., Bragonzoni L., Benvenuti F., Ripamonti C., Benedetti M.G. (2021). Changes in menopausal risk factors in early postmenopausal osteopenic women after 13 months of high-intensity exercise: The randomized controlled ACTLIFE-RCT. Clin. Interv. Aging.

[16] Calvani R., Marini F., Cesari M., Tosato M., Anker S.D., von Haehling S., Miller R.R., Bernabei R., Landi F., Marzetti E. (2015). Biomarkers for physical frailty and sarcopenia: State of the science and future developments. J. Cachexia Sarcopenia Muscle.

[17] Gunendi Z., Ozyemisci-Taskiran O., Demirsoy N. (2008). The effect of 4-week aerobic exercise program on postural balance in postmenopausal women with osteoporosis. Rheumatol. Int..

[18] Valenzuela-Martínez S., Ramírez-Expósito M.J., Carrera-González M.P., Martínez-Martos J.M. (2023). Physiopathology of osteoporosis: Nursing involvement and management. Biomedicines.

[19] Otero M., Esain I., González-Suarez Á.M., Gil S.M. (2017). The effectiveness of a basic exercise intervention to improve strength and balance in women with osteoporosis. Clin. Interv. Aging.

[20] Picorelli A.M., Pereira L.S., Pereira D.S., Felício D., Sherrington C. (2014). Adherence to exercise programs for older people is influenced by program characteristics and personal factors: A systematic review. J. Physiother..

[21] Kirk B., Feehan J., Lombardi G., Duque G. (2020). Muscle, bone, and fat crosstalk: The biological role of myokines, osteokines, and adipokines. Curr. Osteoporos. Rep..

[22] Mancinelli R., Checcaglini F., Coscia F., Gigliotti P., Fulle S., Fanò-Illic G. (2021). Biological aspects of selected myokines in skeletal muscle: Focus on aging. Int. J. Mol. Sci..

[23] Pedersen B.K. (2013). Muscle as a secretory organ. Compr. Physiol..

[24] Li G., Zhang L., Wang D., AIQudsy L., Jiang J.X., Xu H., Shang P. (2019). Muscle-bone crosstalk and potential therapies for sarco-osteoporosis. J. Cell Biochem..

[25] Boström P., Wu J., Jedrychowski M.P., Korde A., Ye L., Lo J.C., Rasbach K.A., Boström E.A., Choi J.H., Long J.Z. (2012). A PGC1-α-dependent myokine that drives brown-fat-like development of white fat and thermogenesis. Nature.

[26] Reza M.M., Subramaniyam N., Sim C.M., Ge X., Sathiakumar D., McFarlane C., Sharma M., Kambadur R. (2017). Irisin is a pro-myogenic factor that induces skeletal muscle hypertrophy and rescues denervation-induced atrophy. Nat. Commun..

[27] Yuan W., Song C. (2022). Crosstalk between bone and other organs. Med. Rev..

[28] Polyzos S.A., Anastasilakis A.D., Efstathiadou Z.A., Makras P., Perakakis N., Kountouras J., Mantzoros C.S. (2018). Irisin in metabolic diseases. Endocrine.

[29] Kim Y.C., Ki S.W., Kim H., Kang S., Kim H., Go G.W. (2023). Recent advances in nutraceuticals for the treatment of sarcopenic obesity. Nutrients.

[30] Morgan E.N., Alsharidah A.S., Mousa A.M., Edrees H.M. (2021). Irisin has a protective role against osteoporosis in ovariectomized rats. Biomed. Res. Int..

[31] Xu L., Shen L., Yu X., Li P., Wang Q., Li C. (2020). Effects of irisin on osteoblast apoptosis and osteoporosis in postmenopausal osteoporosis rats through upregulating Nrf2 and inhibiting NLRP3 inflammasome. Exp. Ther. Med..

[32] Kawao N., Iemura S., Kawaguchi M., Mizukami Y., Takafuji Y., Kaji H. (2021). Role of irisin in effects of chronic exercise on muscle and bone in ovariectomized mice. J. Bone Miner. Metab..

[33] Iemura S., Kawao N., Okumoto K., Akagi M., Kaji H. (2020). Role of irisin in androgen-deficient muscle wasting and osteopenia in mice. J. Bone Miner. Metab..

[34] Luo Y., Ma Y., Qiao X., Zeng R., Cheng R., Nie Y., Li S., Shen X., Yang M., Xu C.C. (2020). Irisin ameliorates bone loss in ovariectomized mice. Climacteric.

[35] Engin-Üstün Y., Çağlayan E.K., Göçmen A.Y., Polat M.F. (2016). Postmenopausal osteoporosis is associated with serum chemerin and irisin but not with apolipoprotein m levels. J. Menopausal Med..

[36] Anastasilakis A.D., Polyzos S.A., Makras P., Gkiomisi A., Bisbinas I., Katsarou A., Filippaios A., Mantzoros C.S. (2014). Circulating irisin is associated with osteoporotic fractures in postmenopausal women with low bone mass but is not affected by either teriparatide or denosumab treatment for 3 months. Osteoporos. Int..

[37] Palermo A., Strollo R., Maddaloni E., Tuccinardi D., D’Onofrio L., Briganti S.I., Defeudis G., De Pascalis M., Lazzaro M.C., Colleluori G. (2015). Irisin is associated with osteoporotic fractures independently of bone mineral density, body composition or daily physical activity. Clin. Endocrinol..

[38] Lu C.W., Wang C.H., Lin Y.L., Kuo C.H., Lai Y.H., Hsu B.G., Tsai J.P. (2021). Serum Irisin level is positively associated with bone mineral density in patients on maintenance hemodialysis. Int. J. Endocrinol..

[39] Zhang J., Huang X., Yu R., Wang Y., Gao C. (2020). Circulating irisin is linked to bone mineral density in geriatric Chinese men. Open Med..

[40] Yan J., Liu H.J., Guo W.C., Yang J. (2018). Low serum concentrations of Irisin are associated with increased risk of hip fracture in Chinese older women. Jt. Bone Spine.

[41] Zhou K., Qiao X., Cai Y., Li A., Shan D. (2019). Lower circulating irisin in middle-aged and older adults with osteoporosis: A systematic review and meta-analysis. Menopause.

[42] Badr Roomi A., Nori W., Mokram Hamed R. (2021). Lower serum irisin levels are associated with increased osteoporosis and oxidative stress in postmenopausal. Rep. Biochem. Mol. Biol..

[43] Liu K., Jing P., Liu Z., Wang Y., Han Z., Wang Y., Zheng Z., Wu Y., Wang T., Li Y. (2021). Serum levels of irisin in postmenopausal women with osteoporotic hip fractures. Cytokine.

[44] Colaianni G., Errede M., Sanesi L., Notarnicola A., Celi M., Zerlotin R., Storlino G., Pignataro P., Oranger A., Pesce V. (2021). Irisin correlates positively with BMD in a cohort of older adult patients and downregulates the senescent marker p21 in osteoblasts. J. Bone Miner. Res..

[45] Park H.S., Kim H.C., Zhang D., Yeom H., Lim S.K. (2019). The novel myokine irisin: Clinical implications and potential role as a biomarker for sarcopenia in postmenopausal women. Endocrine.

[46] Yen C.H., Chang P.S., Chang Y.H., Lin P.T. (2022). Identification of Coenzyme Q10 and skeletal muscle protein biomarkers as potential factors to assist in the diagnosis of sarcopenia. Antioxidants.

[47] Chang J.S., Kim T.H., Nguyen T.T., Park K.S., Kim N., Kong I.D. (2017). Circulating irisin levels as a predictive biomarker for sarcopenia: A cross-sectional community-based study. Geriatr. Gerontol. Int..

[48] Alsaawi T.A., Aldisi D., Abulmeaty M.M.A., Khattak M.N.K., Alnaami A.M., Sabico S., Al-Daghri N.M. (2022). Screening for sarcopenia among elderly arab females: Influence of body composition, lifestyle, irisin, and vitamin D. Nutrients.

[49] Zhou B.N., Zhang Q., Lin X.Y., Hu J., Zhao D.C., Jiang Y., Xing X.P., Li M. (2022). The roles of sclerostin and irisin on bone and muscle of orchiectomized rats. BMC Musculoskelet. Disord..

[50] Wang Y., Gu Y., Huang J., Wu H., Meng G., Zhang Q., Liu L., Zhang S., Wang X., Zhang J. (2022). Serum vitamin D status and circulating irisin levels in older adults with sarcopenia. Front. Nutr..

[51] Choi H.Y., Kim S., Park J.W., Lee N.S., Hwang S.Y., Huh J.Y., Hong H.C., Yoo H.J., Baik S.H., Youn B.S. (2014). Implication of circulating irisin levels with brown adipose tissue and sarcopenia in humans. J. Clin. Endocrinol. Metab..

[52] Baek J.Y., Jang I.Y., Jung H.W., Park S.J., Lee J.Y., Choi E., Lee Y.S., Lee E., Kim B.J. (2022). Serum irisin level is independent of sarcopenia and related muscle parameters in older adults. Exp. Gerontol..

[53] Qaisar R., Karim A., Muhammad T., Shah I., Khan J. (2021). Prediction of sarcopenia using a battery of circulating biomarkers. Sci. Rep..

[54] Lage V.K.D.S., de Paula F.A., Lima L.P., Santos J.N.V., Dos Santos J.M., Viegas Â.A., da Silva G.P., de Almeida H.C., Rodrigues A.L.d.S.N.T., Leopoldino A.A.O. (2022). Plasma levels of myokines and inflammatory markers are related with functional and respiratory performance in older adults with COPD and sarcopenia. Exp. Gerontol..

[55] D’Amuri A., Sanz J.M., Lazzer S., Pišot R., Šimunič B., Biolo G., Zuliani G., Gasparini M., Narici M., Grassi B. (2022). Irisin attenuates muscle impairment during bed rest through muscle-adipose tissue crosstalk. Biology.

[56] Guo M., Yao J., Li J., Zhang J., Wang D., Zuo H., Zhang Y., Xu B., Zhong Y., Shen F. (2023). Irisin ameliorates age-associated sarcopenia and metabolic dysfunction. J. Cachexia Sarcopenia Muscle.

[57] Dozio E., Passeri E., Cardani R., Benedini S., Aresta C., Valaperta R., Corsi Romanelli M., Meola G., Sansone V., Corbetta S. (2017). Circulating irisin is reduced in male patients with type 1 and type 2 myotonic dystrophies. Front. Endocrinol..

[58] Wu Y., Wu Y., Yu J., Zhang Y., Li Y., Fu R., Sun Y., Zhao K., Xiao Q. (2023). Irisin ameliorates D-galactose-induced skeletal muscle fibrosis via the PI3K/Akt pathway. Eur. J. Pharmacol..

[59] Reza M.M., Sim C.M., Subramaniyam N., Ge X., Sharma M., Kambadur R., McFarlane C. (2017). Irisin treatment improves healing of dystrophic skeletal muscle. Oncotarget.

[60] Demir L., Oflazoğlu U. (2023). The relationship between sarcopenia and serum irisin and TNF-α levels in newly diagnosed cancer patients. Support. Care Cancer.

[61] Oflazoglu U., Caglar S., Yılmaz H.E., Önal H.T., Varol U., Salman T., Yildiz Y., Unal S., Guc Z.G., Kucukzeybek Y. (2022). The relationship between sarcopenia detected in newly diagnosed colorectal cancer patients and FGF21, irisin and CRP levels. Eur. Geriatr. Med..

[62] Lee M.J., Lee S.A., Nam B.Y., Park S., Lee S.H., Ryu H.J., Kwon Y.E., Kim Y.L., Park K.S., Oh H.J. (2015). Irisin, a novel myokine is an independent predictor for sarcopenia and carotid atherosclerosis in dialysis patients. Atherosclerosis.

[63] Wu J., Lin S., Guan J., Wu X., Ding M., Shen S. (2023). Prediction of the sarcopenia in peritoneal dialysis using simple clinical information: A machine learning-based model. Semin. Dial..

[64] de Luis D., Primo D., Izaola O., Gómez J.J.L. (2024). Role of irisin and myostatin on sarcopenia in malnourished patients diagnosed with GLIM criteria. Nutrition.

[65] Oguz A., Sahin M., Tuzun D., Kurutas E.B., Ulgen C., Bozkus O., Gul K. (2021). Irisin is a predictor of sarcopenic obesity in type 2 diabetes mellitus: A cross-sectional study. Medicine.

[66] Zhao M., Zhou X., Yuan C., Li R., Ma Y., Tang X. (2020). Association between serum irisin concentrations and sarcopenia in patients with liver cirrhosis: A cross-sectional study. Sci. Rep..

[67] Boga S., Yildirim A.E., Ucbilek E., Koksal A.R., Sisman S.T., Durak I., Sen I., Dogu B., Serin E., Ucbilek A.B. (2022). The effect of sarcopenia and serum myokines on prognosis and survival in cirrhotic patients: A multicenter cross-sectional study. Eur. J. Gastroenterol. Hepatol..

[68] Kukla M., Skladany L., Menżyk T., Derra A., Stygar D., Skonieczna M., Hudy D., Nabrdalik K., Gumprecht J., Marlicz W. (2020). Irisin in liver cirrhosis. J. Clin. Med..

